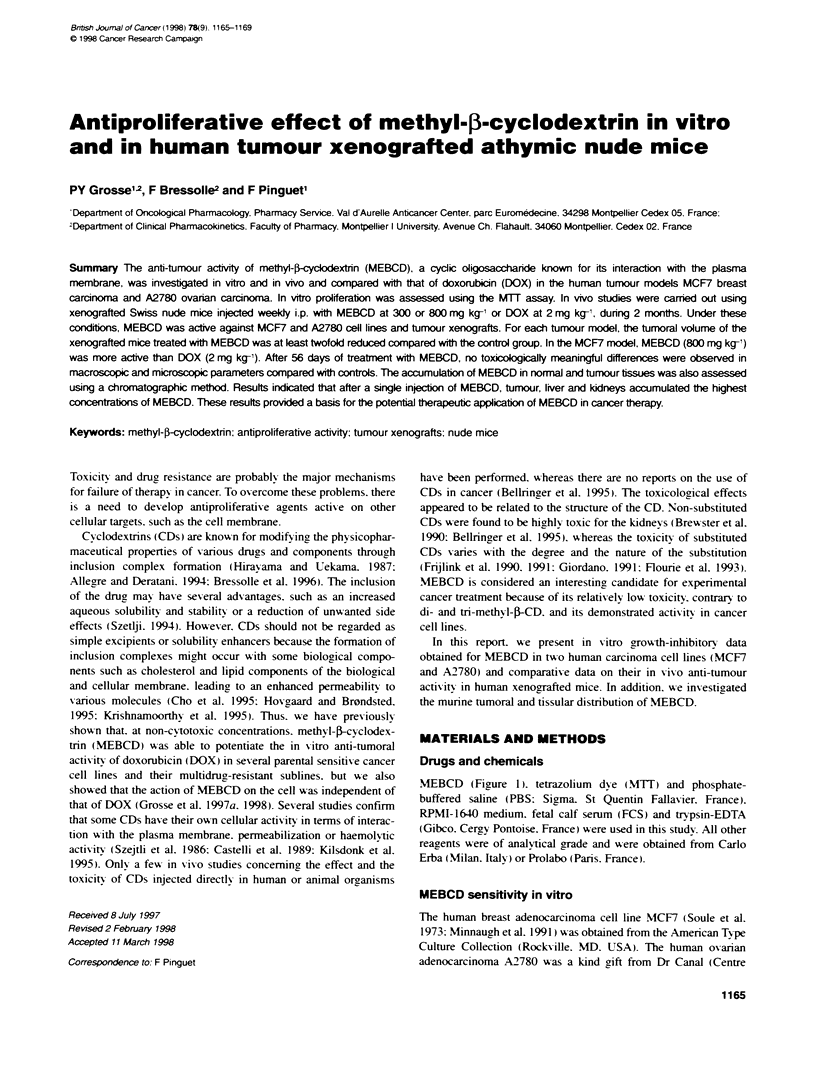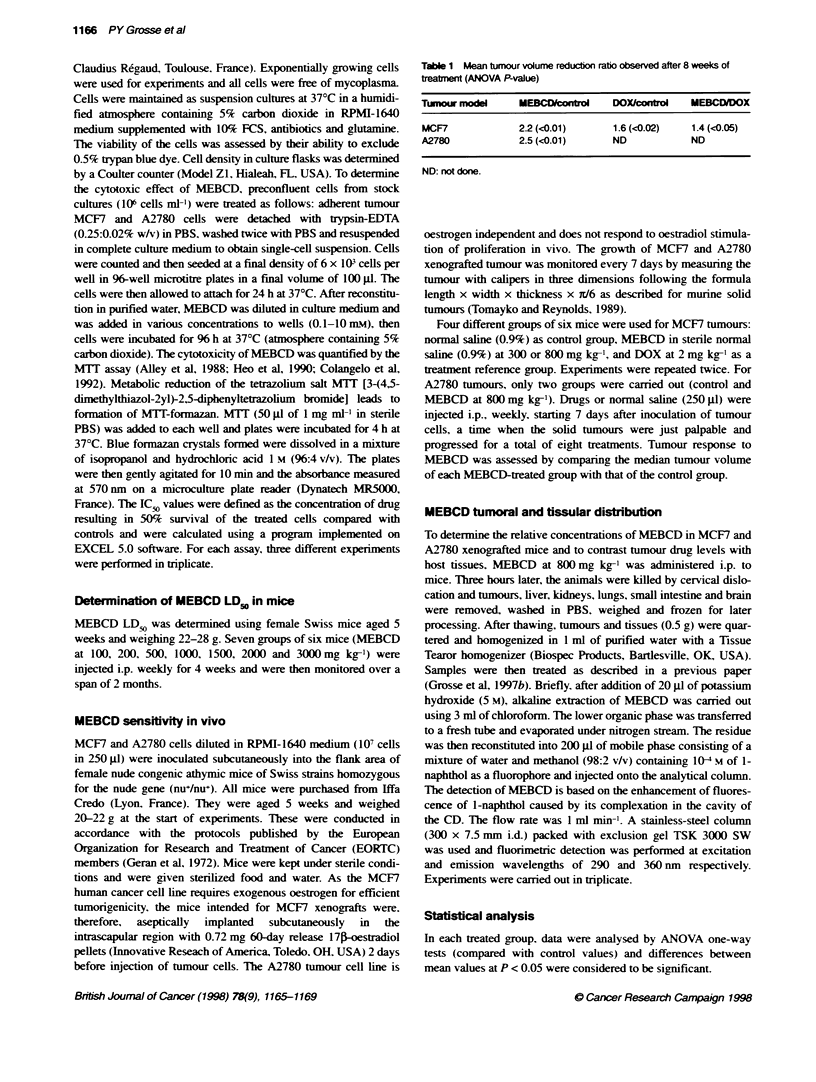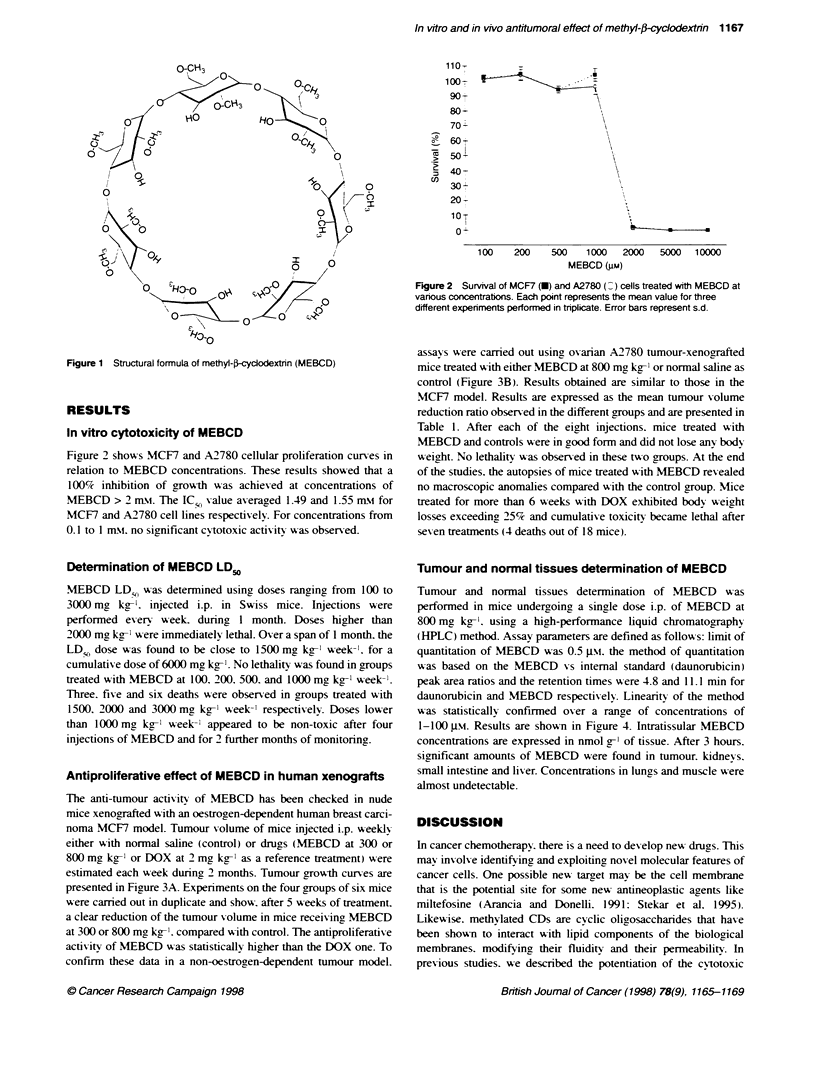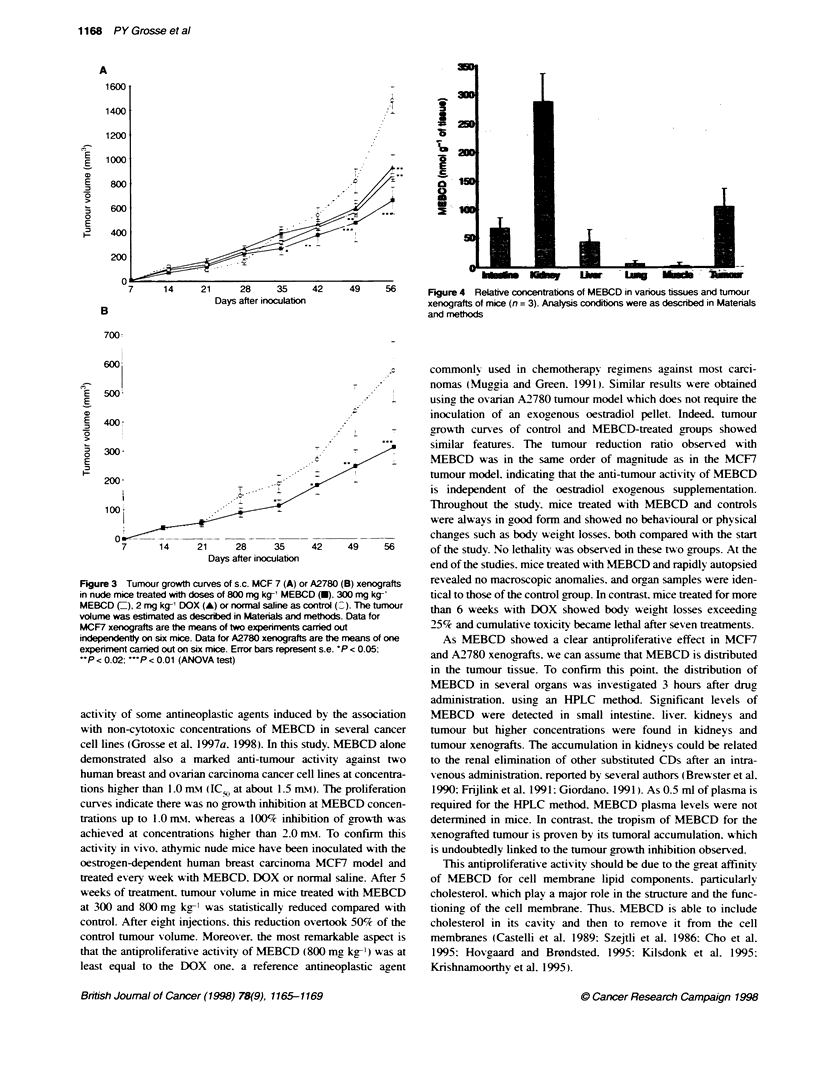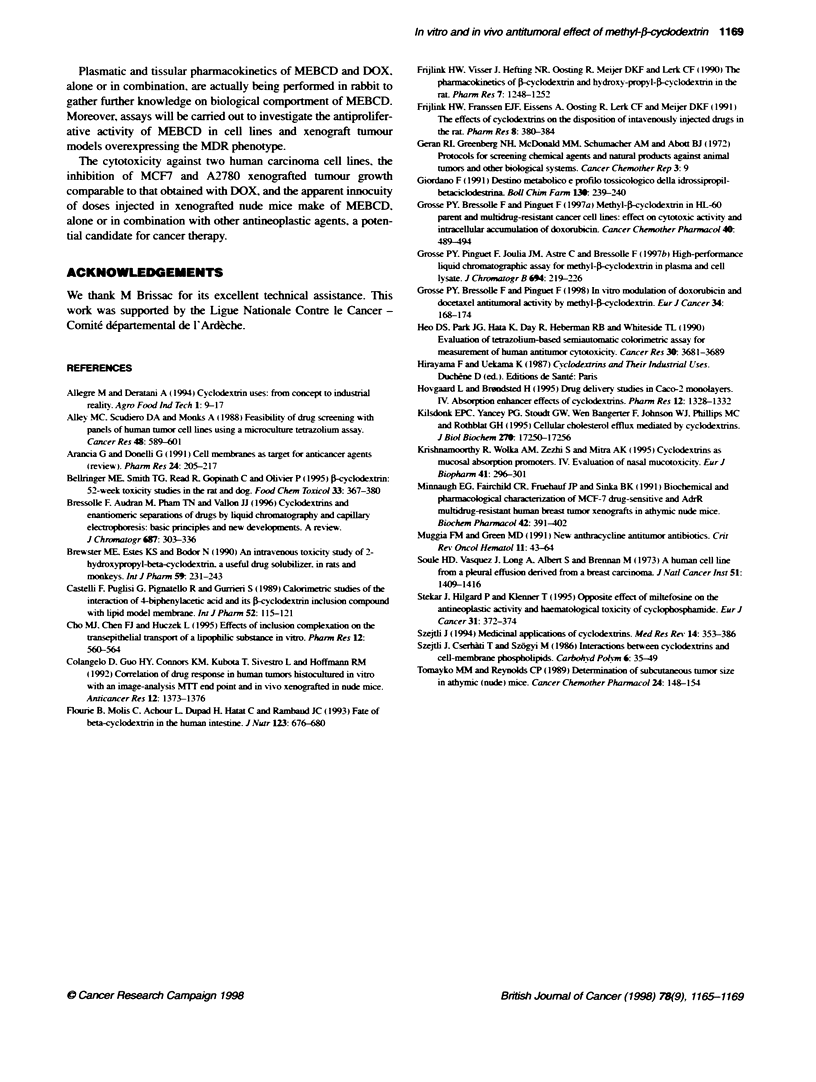# Antiproliferative effect of methyl-beta-cyclodextrin in vitro and in human tumour xenografted athymic nude mice.

**DOI:** 10.1038/bjc.1998.648

**Published:** 1998-11

**Authors:** P. Y. Grosse, F. Bressolle, F. Pinguet

**Affiliations:** Department of Oncological Pharmacology, Pharmacy Service, Val d'Aurelle Anticancer Center, Montpellier, France.

## Abstract

The anti-tumour activity of methyl-beta-cyclodextrin (MEBCD), a cyclic oligosaccharide known for its interaction with the plasma membrane, was investigated in vitro and in vivo and compared with that of doxorubicin (DOX) in the human tumour models MCF7 breast carcinoma and A2780 ovarian carcinoma. In vitro proliferation was assessed using the MTT assay. In vivo studies were carried out using xenografted Swiss nude mice injected weekly i.p. with MEBCD at 300 or 800 mg kg(-1) or DOX at 2 mg kg(-1), during 2 months. Under these conditions, MEBCD was active against MCF7 and A2780 cell lines and tumour xenografts. For each tumour model, the tumoral volume of the xenografted mice treated with MEBCD was at least twofold reduced compared with the control group. In the MCF7 model, MEBCD (800 mg kg(-1)) was more active than DOX (2 mg kg(-1)). After 56 days of treatment with MEBCD, no toxicologically meaningful differences were observed in macroscopic and microscopic parameters compared with controls. The accumulation of MEBCD in normal and tumour tissues was also assessed using a chromatographic method. Results indicated that after a single injection of MEBCD, tumour, liver and kidneys accumulated the highest concentrations of MEBCD. These results provided a basis for the potential therapeutic application of MEBCD in cancer therapy.


British Journal of Cancer (1998) 78(9), 1165–1169
© 1998 Cancer Research Campaign

### Antiproliferative effect of methyl-β-cyclodextrin in vitro and in human tumour xenografted athymic nude mice

**PY Grosse[1,2], F Bressolle[2] and F Pinguet[1]**

[1]Department of Oncological Pharmacology, Pharmacy Service, Val d'Aurelle Anticancer Center, parc Euromédecine, 34298 Montpellier Cedex 05, France; [2]Department of Clinical Pharmacokinetics, Faculty of Pharmacy, Montpellier I University, Avenue Ch. Flahault, 34060 Montpellier, Cedex 02, France

**Summary** The anti-tumour activity of methyl-β-cyclodextrin (MEBCD), a cyclic oligosaccharide known for its interaction with the plasma membrane, was investigated in vitro and in vivo and compared with that of doxorubicin (DOX) in the human tumour models MCF7 breast carcinoma and A2780 ovarian carcinoma. In vitro proliferation was assessed using the MTT assay. In vivo studies were carried out using xenografted Swiss nude mice injected weekly i.p. with MEBCD at 300 or 800 mg $kg^{-1}$ or DOX at 2 mg $kg^{-1}$, during 2 months. Under these conditions, MEBCD was active against MCF7 and A2780 cell lines and tumour xenografts. For each tumour model, the tumoral volume of the xenografted mice treated with MEBCD was at least twofold reduced compared with the control group. In the MCF7 model, MEBCD (800 mg $kg^{-1}$) was more active than DOX (2 mg $kg^{-1}$). After 56 days of treatment with MEBCD, no toxicologically meaningful differences were observed in macroscopic and microscopic parameters compared with controls. The accumulation of MEBCD in normal and tumour tissues was also assessed using a chromatographic method. Results indicated that after a single injection of MEBCD, tumour, liver and kidneys accumulated the highest concentrations of MEBCD. These results provided a basis for the potential therapeutic application of MEBCD in cancer therapy.

**Keywords:** methyl-β-cyclodextrin; antiproliferative activity; tumour xenografts; nude mice

Toxicity and drug resistance are probably the major mechanisms for failure of therapy in cancer. To overcome these problems, there is a need to develop antiproliferative agents active on other cellular targets, such as the cell membrane.

Cyclodextrins (CDs) are known for modifying the physicopharmaceutical properties of various drugs and components through inclusion complex formation (Hirayama and Uekama, 1987; Allegre and Deratani, 1994; Bressolle et al, 1996). The inclusion of the drug may have several advantages, such as an increased aqueous solubility and stability or a reduction of unwanted side effects (Szetlji, 1994). However, CDs should not be regarded as simple excipients or solubility enhancers because the formation of inclusion complexes might occur with some biological components such as cholesterol and lipid components of the biological and cellular membrane, leading to an enhanced permeability to various molecules (Cho et al, 1995; Hovgaard and Brøndsted, 1995; Krishnamoorthy et al, 1995). Thus, we have previously shown that, at non-cytotoxic concentrations, methyl-β-cyclodextrin (MEBCD) was able to potentiate the in vitro anti-tumoral activity of doxorubicin (DOX) in several parental sensitive cancer cell lines and their multidrug-resistant sublines, but we also showed that the action of MEBCD on the cell was independent of that of DOX (Grosse et al, 1997*a*, 1998). Several studies confirm that some CDs have their own cellular activity in terms of interaction with the plasma membrane, permeabilization or haemolytic activity (Szejtli et al, 1986; Castelli et al, 1989; Kilsdonk et al, 1995). Only a few in vivo studies concerning the effect and the toxicity of CDs injected directly in human or animal organisms have been performed, whereas there are no reports on the use of CDs in cancer (Bellringer et al, 1995). The toxicological effects appeared to be related to the structure of the CD. Non-substituted CDs were found to be highly toxic for the kidneys (Brewster et al, 1990; Bellringer et al, 1995), whereas the toxicity of substituted CDs varies with the degree and the nature of the substitution (Frijlink et al, 1990, 1991; Giordano, 1991; Flourie et al, 1993). MEBCD is considered an interesting candidate for experimental cancer treatment because of its relatively low toxicity, contrary to di- and tri-methyl-β-CD, and its demonstrated activity in cancer cell lines.

In this report, we present in vitro growth-inhibitory data obtained for MEBCD in two human carcinoma cell lines (MCF7 and A2780) and comparative data on their in vivo anti-tumour activity in human xenografted mice. In addition, we investigated the murine tumoral and tissular distribution of MEBCD.

*Received 8 July 1997*
*Revised 2 February 1998*
*Accepted 11 March 1998*

*Correspondence to:* F Pinguet

### MATERIALS AND METHODS

### Drugs and chemicals

MEBCD (Figure 1), tetrazolium dye (MTT) and phosphate-buffered saline (PBS; Sigma, St Quentin Fallavier, France), RPMI-1640 medium, fetal calf serum (FCS) and trypsin-EDTA (Gibco, Cergy Pontoise, France) were used in this study. All other reagents were of analytical grade and were obtained from Carlo Erba (Milan, Italy) or Prolabo (Paris, France).

### MEBCD sensitivity in vitro

The human breast adenocarcinoma cell line MCF7 (Soule et al, 1973; Minnaugh et al, 1991) was obtained from the American Type Culture Collection (Rockville, MD, USA). The human ovarian adenocarcinoma A2780 was a kind gift from Dr Canal (Centre

Claudius Régaud, Toulouse, France). Exponentially growing cells were used for experiments and all cells were free of mycoplasma. Cells were maintained as suspension cultures at 37°C in a humidified atmosphere containing 5% carbon dioxide in RPMI-1640 medium supplemented with 10% FCS, antibiotics and glutamine. The viability of the cells was assessed by their ability to exclude 0.5% trypan blue dye. Cell density in culture flasks was determined by a Coulter counter (Model Z1, Hialeah, FL, USA). To determine the cytotoxic effect of MEBCD, preconfluent cells from stock cultures ($10^6$ cells $ml^{-1}$) were treated as follows: adherent tumour MCF7 and A2780 cells were detached with trypsin-EDTA (0.25:0.02% w/v) in PBS, washed twice with PBS and resuspended in complete culture medium to obtain single-cell suspension. Cells were counted and then seeded at a final density of $6 \times 10^3$ cells per well in 96-well microtitre plates in a final volume of 100 µl. The cells were then allowed to attach for 24 h at 37°C. After reconstitution in purified water, MEBCD was diluted in culture medium and was added in various concentrations to wells (0.1–10 mM), then cells were incubated for 96 h at 37°C (atmosphere containing 5% carbon dioxide). The cytotoxicity of MEBCD was quantified by the MTT assay (Alley et al, 1988; Heo et al, 1990; Colangelo et al, 1992). Metabolic reduction of the tetrazolium salt MTT [3-(4,5-dimethylthiazol-2yl)-2,5-diphenyltetrazolium bromide] leads to formation of MTT-formazan. MTT (50 µl of 1 mg $ml^{-1}$ in sterile PBS) was added to each well and plates were incubated for 4 h at 37°C. Blue formazan crystals formed were dissolved in a mixture of isopropanol and hydrochloric acid 1 M (96:4 v/v). The plates were then gently agitated for 10 min and the absorbance measured at 570 nm on a microculture plate reader (Dynatech MR5000, France). The $IC_{50}$ values were defined as the concentration of drug resulting in 50% survival of the treated cells compared with controls and were calculated using a program implemented on EXCEL 5.0 software. For each assay, three different experiments were performed in triplicate.

### Determination of MEBCD $LD_{50}$ in mice

MEBCD $LD_{50}$ was determined using female Swiss mice aged 5 weeks and weighing 22–28 g. Seven groups of six mice (MEBCD at 100, 200, 500, 1000, 1500, 2000 and 3000 mg $kg^{-1}$) were injected i.p. weekly for 4 weeks and were then monitored over a span of 2 months.

### MEBCD sensitivity in vivo

MCF7 and A2780 cells diluted in RPMI-1640 medium ($10^7$ cells in 250 µl) were inoculated subcutaneously into the flank area of female nude congenic athymic mice of Swiss strains homozygous for the nude gene ($nu^+/nu^+$). All mice were purchased from Iffa Credo (Lyon, France). They were aged 5 weeks and weighed 20–22 g at the start of experiments. These were conducted in accordance with the protocols published by the European Organization for Research and Treatment of Cancer (EORTC) members (Geran et al, 1972). Mice were kept under sterile conditions and were given sterilized food and water. As the MCF7 human cancer cell line requires exogenous oestrogen for efficient tumorigenicity, the mice intended for MCF7 xenografts were, therefore, aseptically implanted subcutaneously in the intrascapular region with 0.72 mg 60-day release 17β-oestradiol pellets (Innovative Reseach of America, Toledo, OH, USA) 2 days before injection of tumour cells. The A2780 tumour cell line is oestrogen independent and does not respond to oestradiol stimulation of proliferation in vivo. The growth of MCF7 and A2780 xenografted tumour was monitored every 7 days by measuring the tumour with calipers in three dimensions following the formula length × width × thickness × $\pi/6$ as described for murine solid tumours (Tomayko and Reynolds, 1989).

Four different groups of six mice were used for MCF7 tumours: normal saline (0.9%) as control group, MEBCD in sterile normal saline (0.9%) at 300 or 800 mg $kg^{-1}$, and DOX at 2 mg $kg^{-1}$ as a treatment reference group. Experiments were repeated twice. For A2780 tumours, only two groups were carried out (control and MEBCD at 800 mg $kg^{-1}$). Drugs or normal saline (250 µl) were injected i.p., weekly, starting 7 days after inoculation of tumour cells, a time when the solid tumours were just palpable and progressed for a total of eight treatments. Tumour response to MEBCD was assessed by comparing the median tumour volume of each MEBCD-treated group with that of the control group.

**Table 1** Mean tumour volume reduction ratio observed after 8 weeks of treatment (ANOVA P-value)

| Tumour model | MEBCD/control | DOX/control | MEBCD/DOX |
|---|---|---|---|
| MCF7 | 2.2 (<0.01) | 1.6 (<0.02) | 1.4 (<0.05) |
| A2780 | 2.5 (<0.01) | ND | ND |

ND: not done.

### MEBCD tumoral and tissular distribution

To determine the relative concentrations of MEBCD in MCF7 and A2780 xenografted mice and to contrast tumour drug levels with host tissues, MEBCD at 800 mg $kg^{-1}$ was administered i.p. to mice. Three hours later, the animals were killed by cervical dislocation and tumours, liver, kidneys, lungs, small intestine and brain were removed, washed in PBS, weighed and frozen for later processing. After thawing, tumours and tissues (0.5 g) were quartered and homogenized in 1 ml of purified water with a Tissue Tearor homogenizer (Biospec Products, Bartlesville, OK, USA). Samples were then treated as described in a previous paper (Grosse et al, 1997*b*). Briefly, after addition of 20 µl of potassium hydroxide (5 M), alkaline extraction of MEBCD was carried out using 3 ml of chloroform. The lower organic phase was transferred to a fresh tube and evaporated under nitrogen stream. The residue was then reconstituted into 200 µl of mobile phase consisting of a mixture of water and methanol (98:2 v/v) containing $10^{-4}$ M of 1-naphthol as a fluorophore and injected onto the analytical column. The detection of MEBCD is based on the enhancement of fluorescence of 1-naphthol caused by its complexation in the cavity of the CD. The flow rate was 1 ml $min^{-1}$. A stainless-steel column (300 × 7.5 mm i.d.) packed with exclusion gel TSK 3000 SW was used and fluorimetric detection was performed at excitation and emission wavelengths of 290 and 360 nm respectively. Experiments were carried out in triplicate.

### Statistical analysis

In each treated group, data were analysed by ANOVA one-way tests (compared with control values) and differences between mean values at $P < 0.05$ were considered to be significant.

© Cancer Research Campaign 1998

Figure 1 Structural formula of methyl-β-cyclodextrin (MEBCD)

**Figure 2** Survival of MCF7 (■) and A2780 (□) cells treated with MEBCD at various concentrations. Each point represents the mean value for three different experiments performed in triplicate. Error bars represent s.d.

### RESULTS

### In vitro cytotoxicity of MEBCD

Figure 2 shows MCF7 and A2780 cellular proliferation curves in relation to MEBCD concentrations. These results showed that a 100% inhibition of growth was achieved at concentrations of MEBCD > 2 mM. The $IC_{50}$ value averaged 1.49 and 1.55 mM for MCF7 and A2780 cell lines respectively. For concentrations from 0.1 to 1 mM, no significant cytotoxic activity was observed.

### Determination of MEBCD $LD_{50}$

MEBCD $LD_{50}$ was determined using doses ranging from 100 to 3000 mg $kg^{-1}$, injected i.p. in Swiss mice. Injections were performed every week, during 1 month. Doses higher than 2000 mg $kg^{-1}$ were immediately lethal. Over a span of 1 month, the $LD_{50}$ dose was found to be close to 1500 mg $kg^{-1}$ $week^{-1}$, for a cumulative dose of 6000 mg $kg^{-1}$. No lethality was found in groups treated with MEBCD at 100, 200, 500, and 1000 mg $kg^{-1}$ $week^{-1}$. Three, five and six deaths were observed in groups treated with 1500, 2000 and 3000 mg $kg^{-1}$ $week^{-1}$ respectively. Doses lower than 1000 mg $kg^{-1}$ $week^{-1}$ appeared to be non-toxic after four injections of MEBCD and for 2 further months of monitoring.

### Antiproliferative effect of MEBCD in human xenografts

The anti-tumour activity of MEBCD has been checked in nude mice xenografted with an oestrogen-dependent human breast carcinoma MCF7 model. Tumour volume of mice injected i.p. weekly either with normal saline (control) or drugs (MEBCD at 300 or 800 mg $kg^{-1}$ or DOX at 2 mg $kg^{-1}$ as a reference treatment) were estimated each week during 2 months. Tumour growth curves are presented in Figure 3A. Experiments on the four groups of six mice were carried out in duplicate and show, after 5 weeks of treatment, a clear reduction of the tumour volume in mice receiving MEBCD at 300 or 800 mg $kg^{-1}$, compared with control. The antiproliferative activity of MEBCD was statistically higher than the DOX one. To confirm these data in a non-oestrogen-dependent tumour model, assays were carried out using ovarian A2780 tumour-xenografted mice treated with either MEBCD at 800 mg $kg^{-1}$ or normal saline as control (Figure 3B). Results obtained are similar to those in the MCF7 model. Results are expressed as the mean tumour volume reduction ratio observed in the different groups and are presented in Table 1. After each of the eight injections, mice treated with MEBCD and controls were in good form and did not lose any body weight. No lethality was observed in these two groups. At the end of the studies, the autopsies of mice treated with MEBCD revealed no macroscopic anomalies compared with the control group. Mice treated for more than 6 weeks with DOX exhibited body weight losses exceeding 25% and cumulative toxicity became lethal after seven treatments (4 deaths out of 18 mice).

### Tumour and normal tissues determination of MEBCD

Tumour and normal tissues determination of MEBCD was performed in mice undergoing a single dose i.p. of MEBCD at 800 mg $kg^{-1}$, using a high-performance liquid chromatography (HPLC) method. Assay parameters are defined as follows: limit of quantitation of MEBCD was 0.5 μM, the method of quantitation was based on the MEBCD vs internal standard (daunorubicin) peak area ratios and the retention times were 4.8 and 11.1 min for daunorubicin and MEBCD respectively. Linearity of the method was statistically confirmed over a range of concentrations of 1–100 μM. Results are shown in Figure 4. Intratissular MEBCD concentrations are expressed in nmol $g^{-1}$ of tissue. After 3 hours, significant amounts of MEBCD were found in tumour, kidneys, small intestine and liver. Concentrations in lungs and muscle were almost undetectable.

### DISCUSSION

In cancer chemotherapy, there is a need to develop new drugs. This may involve identifying and exploiting novel molecular features of cancer cells. One possible new target may be the cell membrane that is the potential site for some new antineoplastic agents like miltefosine (Arancia and Donelli, 1991; Stekar et al, 1995). Likewise, methylated CDs are cyclic oligosaccharides that have been shown to interact with lipid components of the biological membranes, modifying their fluidity and their permeability. In previous studies, we described the potentiation of the cytotoxic

**Figure 3** Tumour growth curves of s.c. MCF 7 (**A**) or A2780 (**B**) xenografts in nude mice treated with doses of 800 mg kg$^{-1}$ MEBCD (■), 300 mg kg$^{-1}$ MEBCD (□), 2 mg kg$^{-1}$ DOX (▲) or normal saline as control (○). The tumour volume was estimated as described in Materials and methods. Data for MCF7 xenografts are the means of two experiments carried out independently on six mice. Data for A2780 xenografts are the means of one experiment carried out on six mice. Error bars represent s.e. *$P < 0.05$; **$P < 0.02$; ***$P < 0.01$ (ANOVA test)

activity of some antineoplastic agents induced by the association with non-cytotoxic concentrations of MEBCD in several cancer cell lines (Grosse et al. 1997*a*, 1998). In this study, MEBCD alone demonstrated also a marked anti-tumour activity against two human breast and ovarian carcinoma cancer cell lines at concentrations higher than 1.0 mM ($IC_{50}$ at about 1.5 mM). The proliferation curves indicate there was no growth inhibition at MEBCD concentrations up to 1.0 mM, whereas a 100% inhibition of growth was achieved at concentrations higher than 2.0 mM. To confirm this activity in vivo, athymic nude mice have been inoculated with the oestrogen-dependent human breast carcinoma MCF7 model and treated every week with MEBCD, DOX or normal saline. After 5 weeks of treatment, tumour volume in mice treated with MEBCD at 300 and 800 mg kg$^{-1}$ was statistically reduced compared with control. After eight injections, this reduction overtook 50% of the control tumour volume. Moreover, the most remarkable aspect is that the antiproliferative activity of MEBCD (800 mg kg$^{-1}$) was at least equal to the DOX one, a reference antineoplastic agent

**Figure 4** Relative concentrations of MEBCD in various tissues and tumour xenografts of mice ($n = 3$). Analysis conditions were as described in Materials and methods commonly used in chemotherapy regimens against most carcinomas (Muggia and Green, 1991). Similar results were obtained using the ovarian A2780 tumour model which does not require the inoculation of an exogenous oestradiol pellet. Indeed, tumour growth curves of control and MEBCD-treated groups showed similar features. The tumour reduction ratio observed with MEBCD was in the same order of magnitude as in the MCF7 tumour model, indicating that the anti-tumour activity of MEBCD is independent of the oestradiol exogenous supplementation. Throughout the study, mice treated with MEBCD and controls were always in good form and showed no behavioural or physical changes such as body weight losses, both compared with the start of the study. No lethality was observed in these two groups. At the end of the studies, mice treated with MEBCD and rapidly autopsied revealed no macroscopic anomalies, and organ samples were identical to those of the control group. In contrast, mice treated for more than 6 weeks with DOX showed body weight losses exceeding 25% and cumulative toxicity became lethal after seven treatments.

As MEBCD showed a clear antiproliferative effect in MCF7 and A2780 xenografts, we can assume that MEBCD is distributed in the tumour tissue. To confirm this point, the distribution of MEBCD in several organs was investigated 3 hours after drug administration, using an HPLC method. Significant levels of MEBCD were detected in small intestine, liver, kidneys and tumour but higher concentrations were found in kidneys and tumour xenografts. The accumulation in kidneys could be related to the renal elimination of other substituted CDs after an intravenous administration, reported by several authors (Brewster et al, 1990; Frijlink et al, 1991; Giordano, 1991). As 0.5 ml of plasma is required for the HPLC method, MEBCD plasma levels were not determined in mice. In contrast, the tropism of MEBCD for the xenografted tumour is proven by its tumoral accumulation, which is undoubtedly linked to the tumour growth inhibition observed.

This antiproliferative activity should be due to the great affinity of MEBCD for cell membrane lipid components, particularly cholesterol, which play a major role in the structure and the functioning of the cell membrane. Thus, MEBCD is able to include cholesterol in its cavity and then to remove it from the cell membranes (Castelli et al, 1989; Szejtli et al, 1986; Cho et al, 1995; Hovgaard and Brøndsted, 1995; Kilsdonk et al, 1995; Krishnamoorthy et al, 1995).

© Cancer Research Campaign 1998

Plasmatic and tissular pharmacokinetics of MEBCD and DOX, alone or in combination, are actually being performed in rabbit to gather further knowledge on biological comportment of MEBCD. Moreover, assays will be carried out to investigate the antiproliferative activity of MEBCD in cell lines and xenograft tumour models overexpressing the MDR phenotype.

The cytotoxicity against two human carcinoma cell lines, the inhibition of MCF7 and A2780 xenografted tumour growth comparable to that obtained with DOX, and the apparent innocuity of doses injected in xenografted nude mice make of MEBCD, alone or in combination with other antineoplastic agents, a potential candidate for cancer therapy.

### ACKNOWLEDGEMENTS

We thank M Brissac for its excellent technical assistance. This work was supported by the Ligue Nationale Contre le Cancer – Comité départemental de l'Ardèche.

### REFERENCES

Allegre M and Deratani A (1994) Cyclodextrin uses: from concept to industrial reality. *Agro Food Ind Tech* **1**: 9–17

Alley MC, Scudiero DA and Monks A (1988) Feasibility of drug screening with panels of human tumor cell lines using a microculture tetrazolium assay. *Cancer Res* **48**: 589–601

Arancia G and Donelli G (1991) Cell membranes as target for anticancer agents (review). *Pharm Res* **24**: 205–217

Bellringer ME, Smith TG, Read R, Gopinath C and Olivier P (1995) β-cyclodextrin: 52-week toxicity studies in the rat and dog. *Food Chem Toxicol* **33**: 367–380

Bressolle F, Audran M, Pham TN and Vallon JJ (1996) Cyclodextrins and enantiomeric separations of drugs by liquid chromatography and capillary electrophoresis: basic principles and new developments. A review. *J Chromatogr* **687**: 303–336

Brewster ME, Estes KS and Bodor N (1990) An intravenous toxicity study of 2-hydroxypropyl-beta-cyclodextrin, a useful drug solubilizer, in rats and monkeys. *Int J Pharm* **59**: 231–243

Castelli F, Puglisi G, Pignatello R and Gurrieri S (1989) Calorimetric studies of the interaction of 4-biphenylacetic acid and its β-cyclodextrin inclusion compound with lipid model membrane. *Int J Pharm* **52**: 115–121

Cho MJ, Chen FJ and Huczek L (1995) Effects of inclusion complexation on the transepithelial transport of a lipophilic substance in vitro. *Pharm Res* **12**: 560–564

Colangelo D, Guo HY, Connors KM, Kubota T, Sivestro L and Hoffmann RM (1992) Correlation of drug response in human tumors histocultured in vitro with an image-analysis MTT end point and in vivo xenografted in nude mice. *Anticancer Res* **12**: 1373–1376

Flourie B, Molis C, Achour L, Dupad H, Hatat C and Rambaud JC (1993) Fate of beta-cyclodextrin in the human intestine. *J Nutr* **123**: 676–680

Frijlink HW, Visser J, Hefting NR, Oosting R, Meijer DKF and Lerk CF (1990) The pharmacokinetics of β-cyclodextrin and hydroxy-propyl-β-cyclodextrin in the rat. *Pharm Res* **7**: 1248–1252

Frijlink HW, Franssen EJF, Eissens A, Oosting R, Lerk CF and Meijer DKF (1991) The effects of cyclodextrins on the disposition of intavenously injected drugs in the rat. *Pharm Res* **8**: 380–384

Geran RI, Greenberg NH, McDonald MM, Schumacher AM and Abott BJ (1972) Protocols for screening chemical agents and natural products against animal tumors and other biological systems. *Cancer Chemother Rep* **3**: 9

Giordano F (1991) Destino metabolico e profilo tossicologico della idrossipropil-betaciclodestrina. *Boll Chim Farm* **130**: 239–240

Grosse PY, Bressolle F and Pinguet F (1997*a*) Methyl-β-cyclodextrin in HL-60 parent and multidrug-resistant cancer cell lines: effect on cytotoxic activity and intracellular accumulation of doxorubicin. *Cancer Chemother Pharmacol* **40**: 489–494

Grosse PY, Pinguet F, Joulia JM, Astre C and Bressolle F (1997*b*) High-performance liquid chromatographic assay for methyl-β-cyclodextrin in plasma and cell lysate. *J Chromatogr B* **694**: 219–226

Grosse PY, Bressolle F and Pinguet F (1998) In vitro modulation of doxorubicin and docetaxel antitumoral activity by methyl-β-cyclodextrin. *Eur J Cancer* **34**: 168–174

Heo DS, Park JG, Hata K, Day R, Heberman RB and Whiteside TL (1990) Evaluation of tetrazolium-based semiautomatic colorimetric assay for measurement of human antitumor cytotoxicity. *Cancer Res* **30**: 3681–3689

Hirayama F and Uekama K (1987) *Cyclodextrins and Their Industrial Uses*. Duchêne D (ed.). Editions de Santé: Paris

Hovgaard L and Brøndsted H (1995) Drug delivery studies in Caco-2 monolayers. IV. Absorption enhancer effects of cyclodextrins. *Pharm Res* **12**: 1328–1332

Kilsdonk EPC, Yancey PG, Stoudt GW, Wen Bangerter F, Johnson WJ, Phillips MC and Rothblat GH (1995) Cellular cholesterol efflux mediated by cyclodextrins. *J Biol Biochem* **270**: 17250–17256

Krishnamoorthy R, Wolka AM, Zezhi S and Mitra AK (1995) Cyclodextrins as mucosal absorption promoters. IV. Evaluation of nasal mucotoxicity. *Eur J Biopharm* **41**: 296–301

Minnaugh EG, Fairchild CR, Fruehauf JP and Sinka BK (1991) Biochemical and pharmacological characterization of MCF-7 drug-sensitive and AdrR multidrug-resistant human breast tumor xenografts in athymic nude mice. *Biochem Pharmacol* **42**: 391–402

Muggia FM and Green MD (1991) New anthracycline antitumor antibiotics. *Crit Rev Oncol Hematol* **11**: 43–64

Soule HD, Vasquez J, Long A, Albert S and Brennan M (1973) A human cell line from a pleural effusion derived from a breast carcinoma. *J Natl Cancer Inst* **51**: 1409–1416

Stekar J, Hilgard P and Klenner T (1995) Opposite effect of miltefosine on the antineoplastic activity and haematological toxicity of cyclophosphamide. *Eur J Cancer* **31**: 372–374

Szejtli J (1994) Medicinal applications of cyclodextrins. *Med Res Rev* **14**: 353–386

Szejtli J, Cserhàti T and Szögyi M (1986) Interactions between cyclodextrins and cell-membrane phospholipids. *Carbohyd Polym* **6**: 35–49

Tomayko MM and Reynolds CP (1989) Determination of subcutaneous tumor size in athymic (nude) mice. *Cancer Chemother Pharmacol* **24**: 148–154

© Cancer Research Campaign 1998